# The distribution of the vascular plants on the North Frisian Island, Amrum

**DOI:** 10.3897/BDJ.2.e1108

**Published:** 2014-06-10

**Authors:** Quentin John Groom

**Affiliations:** † Agentschap Plantentuin Meise, Meise, Belgium

**Keywords:** Amrum, vascular plants, plant survey

## Abstract

Amrum is a small barrier island on the north-west coast of Germany. The distribution of vascular plants was examined by surveying their 1km^2^ grid square occupancy across the whole island. These data were used in a study on the recent vegetation change in the island. These data include 3786 observations of 450 taxa collected in two surveys in 2007 and 2008.

## Introduction

Amrum is a small barrier island off the coast of Schleswig-Holstein, Germany. It covers an area of about 20 km^2^, though considerable areas of saltmarsh vegetation are exposed at low tide. Amrum has been the subject of many botanical surveys and this survey continues that tradition (Christiansen 1961, Türk 1994, Petersen 2000, von Seemen 1899, Schiøtz 1860).

**Figure 1.**
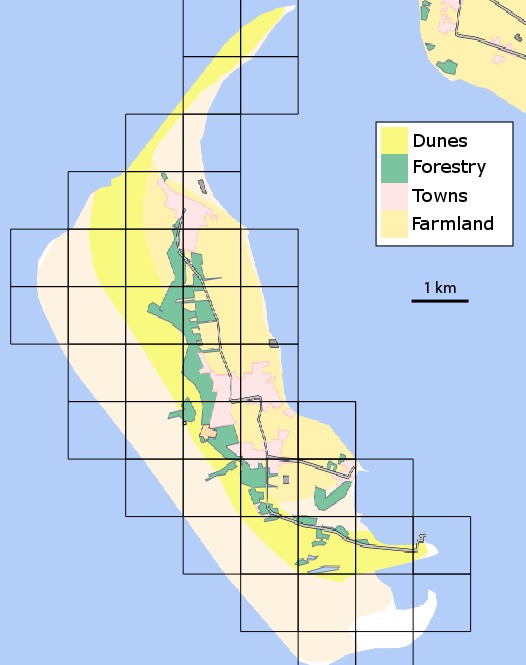
A map of Amrum showing the main landscape features and the 1km^2^ grid used for the survey. The grid is based upon the spatial reference system EPSG:31467 - DHDN / Gauss-Kruger zone 3.

Amrum is approximately 10 km long and 3 km wide at its widest point (Fig. 1). Its main habitats run in north-south bands along the island. In the west are open sand flats with a sparse fore-dune vegetation, moving eastward there is a complete dune succession ending in conifer plantations. Beyond the plantations is arable farmland and grazing. Along the eastern shore are mainly saltmarshes and mudflats. Other important habitats include freshwater ponds, marshes and ditches, heathland, urban areas and the seawall.

These data were used in a study of change in the islands flora (Groom 2010). They are published in the hope that they will help future studies.

## Sampling methods

### Study extent

The survey covered all vegetated areas of the island were access was permitted. In fact, this covers most of the island, however, some nesting bird colonies and private gardens are completely inaccessible.

### Sampling description

The biodiversity of each 1 km^2^ varies considerable so there was no set time limit for surveying a particular square. Grid squares were surveyed as intensively as considered necessary to find all but the rarest species. Aerial photographs, maps, previous surveys and local experts were all used to direct surveying to all potentially important habitats. The grid is based upon the spatial reference system EPSG:31467 - DHDN / Gauss-Kruger zone 3 (Fig. 1).

### Quality control

Some *Salix* specimens where identified by R.D. Meikle.

## Geographic coverage

### Description

Amrum is one of the North Frisian Islands, Schleswig-Holstein, Germany. Its western coast faces the North Sea and the eastern coast faces the Wadden Sea (Fig. 1).

### Coordinates

54° 36' 22.9602" and 54° 42' 44.1936" Latitude; 8° 17' 26.5308" and 8° 24' 12.6138" Longitude.

## Taxonomic coverage

### Description

The study covers all vascular plants growing in the wild. Obviously cultivated plants were excluded, though trees planted for forestry were included as these form so much of the island's habitats. Some examples of natural habitats on Amrum are illustrated (Fig. 2).

## Temporal coverage

**Data range:** 2008 7 05 – 2008 7 20; 2007 8 17 – 2007 8 31.

## Usage rights

### Use license

Creative Commons CCZero

## Data resources

### Data package title

Vascular plant survey of Amrum 2007-2008

### Resource link

http://www.gbif.org/dataset/e6fb3d46-977a-4deb-99be-e8493024feb2

### Number of data sets

1

### Data set 1.

#### Data set name

Survey of the whole island

#### Data format

Darwin Core archive

#### Number of columns

43

#### Character set

UTF-8

#### Data format version

1

#### Description

**Data set 1.** 

Column label	Column description
genus	The genera of the taxon.
specificEpithet	The specific epithet of the taxon.
taxonRank	The taxonomic rank of the name.
infraspecificEpithet	The infraspecific name of the taxon, if identified to this rank.
scientificName	full scientific names, written out as a string with authority.
kingdom	The taxonomic kingdom to which the organism belongs.
family	The taxonomic family to which the organism belongs.
vernacularName	The English common name of the taxon.
nomenclaturalCode	International Code of Nomenclature for the name.
identifiedBy	The identifier of the specimen.
recordedBy	The person who found the specimen.
language	The language used in the observation details.
rights	The rights given to the use of these data.
rightsHolder	The person to whom these right belong.
datasetID	A unique identifier to this dataset.
datasetName	The name of this dataset.
basisOfRecord	How the observation was made.
occurrenceStatus	An indication of the about the presence or absence of a taxon at a location.
individualCount	The number of individuals observed. If left blank the taxon was observed, but not counted.
eventDate	The date on which the observation occurred.
month	The month in which the observation occurred.
year	The year in which the observation occurred.
occurrenceRemarks	Additional notes on the observation.
establishmentMeans	How the taxon became established at the location. Frequently used where the taxon was planted.
occurrenceID	A unique identifier to the observation.
locality	A textual description of the precise locality.
higherGeography	A textual description of the region in which the observation occurred.
islandGroup	The name of the island group in which the observation occurred.
island	The name of the island in which the observation occurred.
country	The name of the country in which the observation occurred.
countryCode	The ISO 3166-1-alpha-2 country code.
stateProvince	The first-level administrative subdivision of the country in which the observation occurred.
continent	The continent in which the observation occurred.
verbatimCoordinates	The geographic coordinates of the observation written out.
verbatimSRS	The spatial reference system of the verbatim coordinates.
footprintWKT	A Well-Known Text (WKT) representation of the shape that defines the location.
footprintSRS	The spatial reference system of the footprint WKT coordinates.
habitat	A textual description of the habitat in which the taxon was observed.
coordinateUncertaintyInMeters	The limit of the distance (in meters) from the given coordinates describing a circle within which the locality must lie.
decimalLongitude	The longitude of the location the observation was made, in decimal degrees.
decimalLatitude	The latitude of the location the observation was made, in decimal degrees.
geodeticDatum	The geodetic datum to which the latitude and longitude refer.
pointRadiusSpatialFit	The ratio of the area of the point-radius to the actual area of the spatial representation of the location

## Supplementary Material

Supplementary material 1 The distribution of the vascular plants on the North Frisian Island, Amrum Data type: occurrences
Brief description: A Darwin Core Archive of vascular plant occurrences from the island of Amrum, Germany recorded in 2007 and 2008
File: oo_6541.zip Quentin J. Groom

## Figures and Tables

**Figure 2a.**
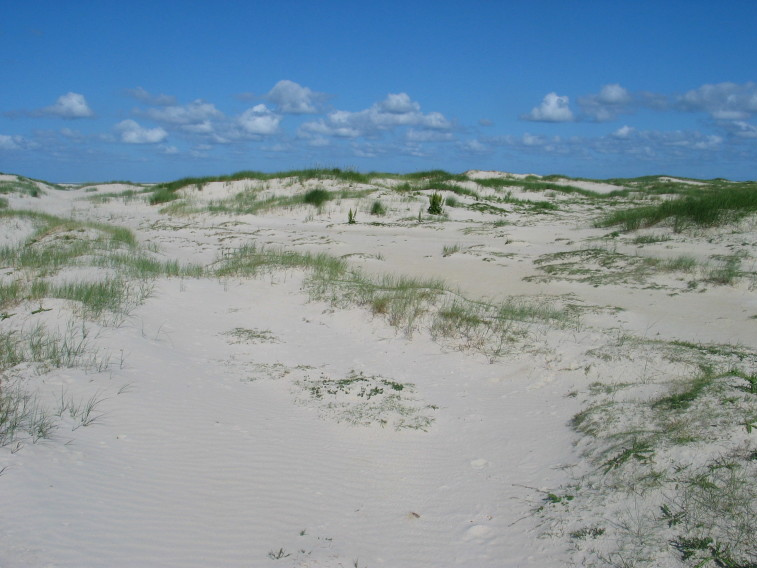
Mobile foredunes containing *Ammophila arenaria*, *Rumex crispus*, *Sonchus arvensis* and *Elytrigia juncea*.

**Figure 2b.**
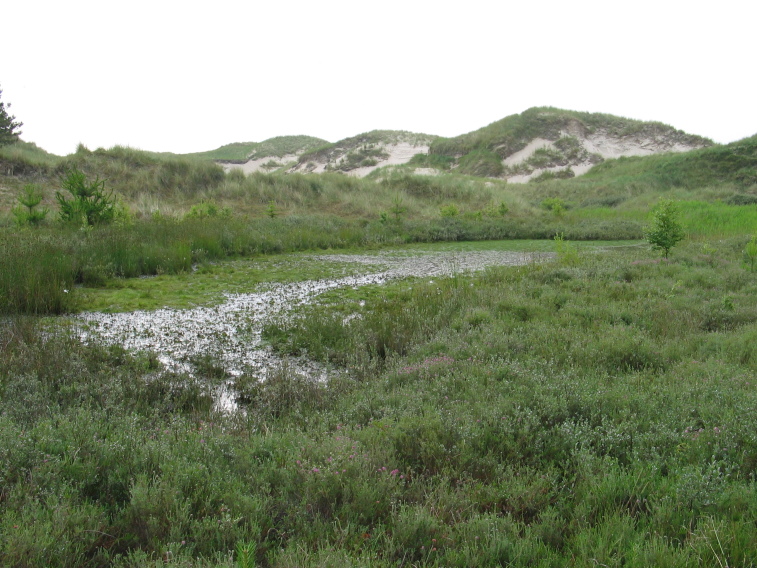
A mature wet dune slack, habitat to *Erica tetralix* and *Eriophorum angustifolium*.

**Figure 2c.**
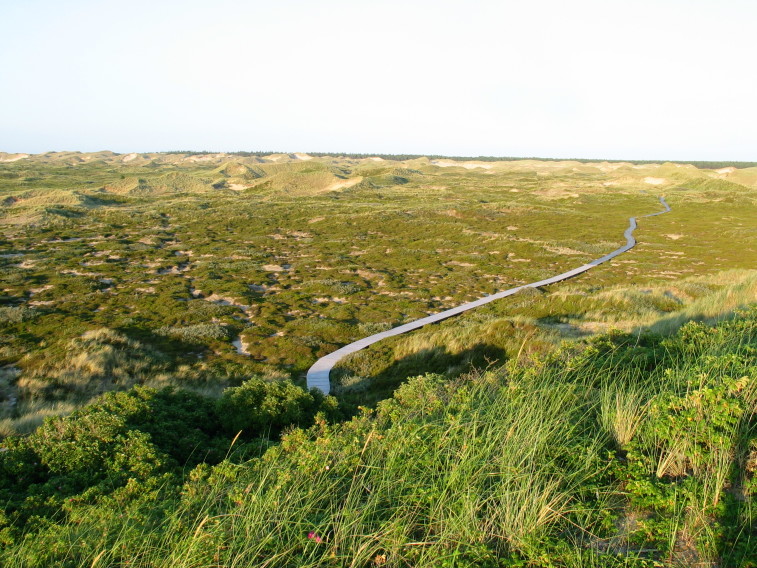
*
Calluna vulgaris* heath on stabilized dunes, taken from the lighthouse called the Quermarkenfeuer (54°40'9.25"N, 8°18'30.84"E).

**Figure 2d.**
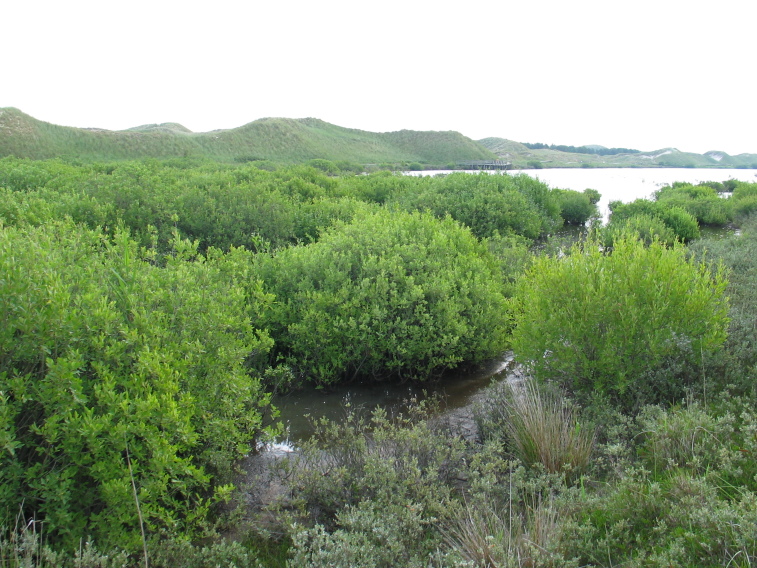
*
Salix cinerea* carr at the western end of the Nehrungssee (54°37'22.77"N, 8°22'0.42"E).

**Figure 2e.**
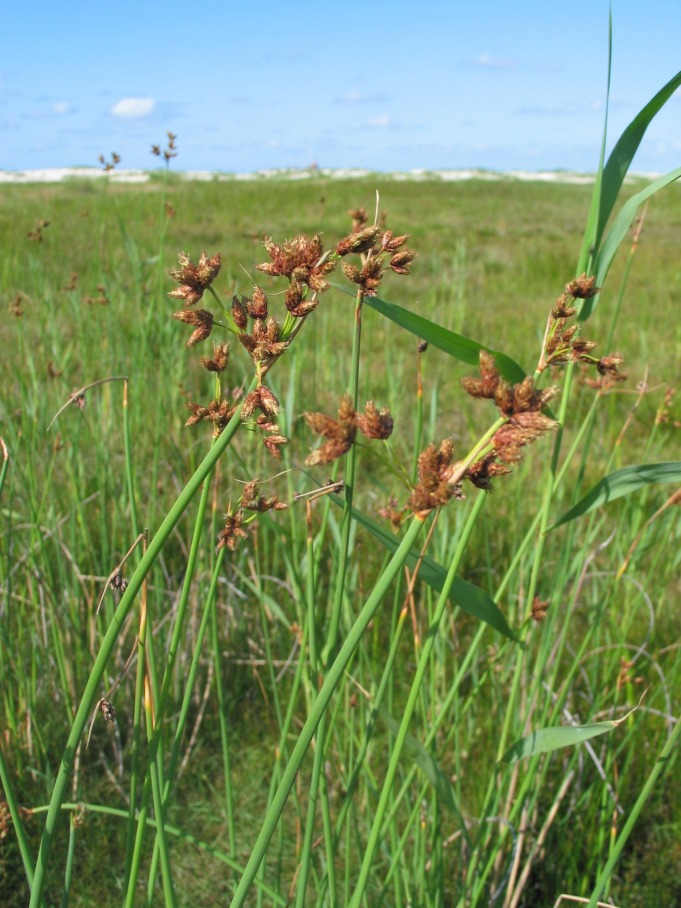
*
Bolboschoenus maritimus*

**Figure 2f.**
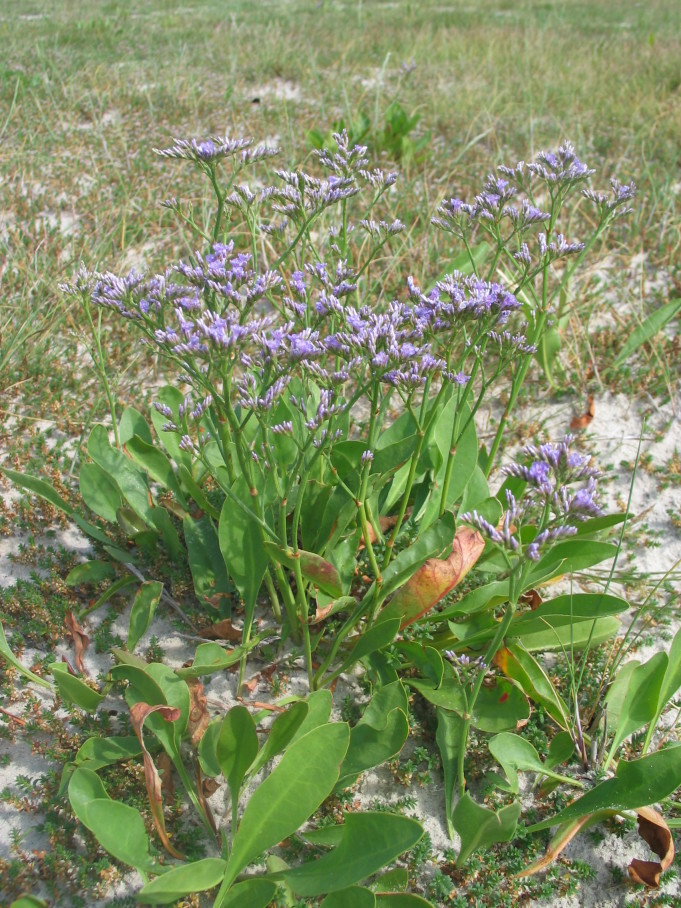
*
Limonium vulgare*
